# Variations in the Formation of the Spinal Accessory Nerve: Spinal Root

**DOI:** 10.7759/cureus.99462

**Published:** 2025-12-17

**Authors:** Priyanka N Sharma, Hetal Vaishnani, Kinjal V Jethva, Manoj M Kulkarni, Achleshwar R Gandotra

**Affiliations:** 1 Anatomy, Smt. B. K. Shah Medical Institute and Research Centre, Sumandeep Vidyapeeth (Deemed to be University), Vadodara, IND

**Keywords:** accessory nerve, cervical segment, cranial root, dorsal, dorsal root, spinal accessory nerve, spinal root, sternocleidomastoid, trapezius

## Abstract

Introduction

The spinal accessory nerve (SAN), traditionally recognized as the 11th cranial nerve, consists of a cranial root arising from the medulla oblongata and a spinal root arising from the upper cervical spinal cord. Despite more than a century of investigation, significant uncertainty persists regarding its precise cervical segmental origins, segmental contribution, and dorsal rootlet pattern, which are critical for safe surgical navigation during skull base procedures, posterior cervical exposure, and neck dissections. This study aims to document the length, segmental origins, and dorsal rootlet contributions of the spinal component of the spinal accessory nerve in adult cadavers.

Methods

Thirty formalin-fixed adult cadavers (60 sides) underwent standardized posterior craniovertebral dissection. The spinal portion of the spinal accessory nerve was exposed from the caudal cervical segment to the jugular foramen of the skull. Rootlets from C1 to C7 were identified, and the spinal root length was measured using a digital Vernier caliper. The segmental and dorsal spinal rootlet contributions to the formation of the spinal accessory nerve have been documented. Data were statistically analyzed using descriptive statistics, Shapiro-Wilk tests, paired t-tests, Welch’s t-tests, chi-square tests, and Wilcoxon signed-rank tests.

Results

The spinal accessory nerve measured 56.8 ± 17.0 mm on the right and 58.5 ± 13.1 mm on the left, with no significant side differences. The spinal segments that provide rootlets for the formation of the spinal accessory nerve were found to be the C1-C7 segments. The most frequent formation patterns were C1-C4 (right) and C1-C5 (left) patterns. Across all 60 nerves, the highest total dorsal rootlet (n = 350) contribution arose from C2 (38.3%), followed by C3 (22.6%) and C1 (19.7%). The contributions of C5-C7 were minimal. The chi-square test comparing the segmental pattern distribution between the sides was not significant.

Conclusion

Previous literature has not reported a detailed measurement of the accessory nerve length. In this study, the spinal accessory nerve was measured as 56.8 ± 17.0 mm on the right side and 58.5 ± 13.1 mm on the left side, with no significant difference observed between the two sides. The spinal accessory nerve receives rootlets from the cervical segments C1-C7. These findings refine the anatomical understanding of spinal accessory nerve organization and underscore the necessity of incorporating such variability into surgical planning to mitigate iatrogenic injury in cervical and skull base procedures.

## Introduction

The spinal accessory nerve (SAN), also known as cranial nerve XI or the nervus accessorius, is a complex neuroanatomical structure. It was initially described and illustrated as a distinct cranial nerve by Thomas Willis in 1664, who termed it “accessory” because of its additional root originating from the spinal cord [1,2]. The SAN comprises both cranial and spinal roots. The cranial root is thought to originate from three to six small rootlets on the dorsolateral surface of the medulla oblongata, specifically from the caudal region of the nucleus ambiguus [3-9]. In contrast, some authors have reported that the spinal portion arises from the upper five or six rootlets that emerge from the spinal nucleus within the lateral gray matter of the cervical spinal levels C1 to C5 or C6 [3-5,7-12], and in some cases, it is found at C7 [13-15] or T1 [16]. These spinal rootlets ascend through the spinal canal, enter the posterior cranial fossa via the foramen magnum, and are situated posterior to the vertebral artery and dorsal to the denticulate ligaments. They subsequently merge with the cranial root to form a unified trunk of the accessory nerves. The trunk ascends and moves laterally to traverse the jugular foramen. As it passes through the jugular foramen, it is encased in a dural sheath, travelling laterally to the vagus nerve and anterior to the internal jugular vein (IJV). While passing through the IJV, the accessory nerve establishes connections with the vagus nerve through its internal ramus or pars vagalis with the superior ganglion of the vagus nerve [1,17].

The accessory nerve is unique in that it exclusively transmits motor signals to the sternocleidomastoid (SCM), whereas the C2 and C3 cervical nerve fibers carry proprioceptive signals away from it. The extension of the trapezius muscle represents the terminal trunk of the accessory nerve [5,18-21]. Iatrogenic injury to the accessory nerve is frequently associated with lymph node biopsies performed by general surgeons, as damage at this point can lead to shoulder syndrome, characterized by shoulder pain and drooping, impaired shoulder abduction, and atrophy of the SCM and trapezius muscles, along with compensatory hypertrophy of other shoulder muscles and limited active coronal plane abduction [5]. The accessory nerve can be used as a transferable nerve in neurotization and reinnervation procedures [18]. Most anatomy textbooks indicate that the spinal rootlets of the accessory nerve extend down to the fifth or sixth cervical segment [8]. Despite the extensive literature, variations remain underreported in many populations, including the Indian subcontinent. Therefore, the present study investigated the spinal component of the SAN, with an emphasis on rootlet origin, segmental contribution, and morphometric characteristics. This information could be crucial for neurosurgeons during surgical procedures in this area or for those interpreting the imaging of the posterior cranial fossa and employing micro-operative techniques to address lesions of the foramen magnum [15].

## Materials and methods

Thirty formalin-fixed adult cadavers (60 sides; 15 men and 15 women) with a mean age at death of 77 years underwent SAN dissection from its cranial and spinal origins to its emergence in the jugular foramen. The present study was conducted at the Department of Anatomy of a teaching medical institute in Gujarat, India. Ethical approval for the present study was obtained from the Sumandeep Vidyapeeth Institutional Ethics Committee (reference letter number: SVIEC/OW/MEDI/PHD/18005).

The cervical region was dorsally dissected in 60 specimens without any previous spinal surgical procedures, spinal cord pathology, or nontraumatic pathology at the medical institute in India. The occipital bone and posterior neural arch of the cervical vertebrae from C1 to C7 were excised, and the underlying dura mater covering the craniocervical junction and cervical spine was incised. The arachnoid mater was carefully cleaned to ensure that the denticulate ligaments remained intact. The visible topographical relationship between the vessels and denticulate ligaments was observed with the help of a surgical magnification loupe with 4× magnification, and the formation and course of the SAN were followed on both sides from its most caudal point to the jugular foramen, and the cervical segment contribution and rootlets were followed. The length of the SAN spinal root was measured from the caudal origin of the cervical segment to the jugular foramen, where it connects with the cranial root to form a common SAN trunk. The length of the nerve was measured using a digital Vernier caliper. To further verify these findings, a specimen was excised from the lower medulla oblongata to the cervical spinal cord and mounted on a wax block for further analysis. All observations were recorded in a standardized format, identifying the most caudal level and counting the number of dorsal rootlets contributing to the formation of the SAN at each cervical segment on both sides and in both sexes.

The data were compiled into a comprehensive database for statistical analysis. Statistical analyses were performed using Microsoft Excel (Microsoft Corp., Redmond, WA) and SPSS version 26 (IBM Corp., Armonk, NY). Descriptive statistics (mean, standard deviation, range, and 95% confidence interval) were calculated for the length of the SAN. The normality of continuous variables was assessed using the Shapiro-Wilk test. Side differences in SAN length were evaluated using a paired t-test for normally distributed data, and sex-wise comparisons were performed using Welch’s t-test, assuming unequal variances. The segmental contributions of the cervical spinal nerves to SAN formation and patterns of dorsal rootlet contribution were summarized as frequencies and percentages. Sidewise differences in categorical distributions were analyzed using the chi-square test. The total number of dorsal rootlets from C1 to C7 that formed the SAN on each side was compared using the Wilcoxon signed-rank test when normality assumptions were not met. Statistical significance was set at p < 0.05.

## Results

**Figure 1:**
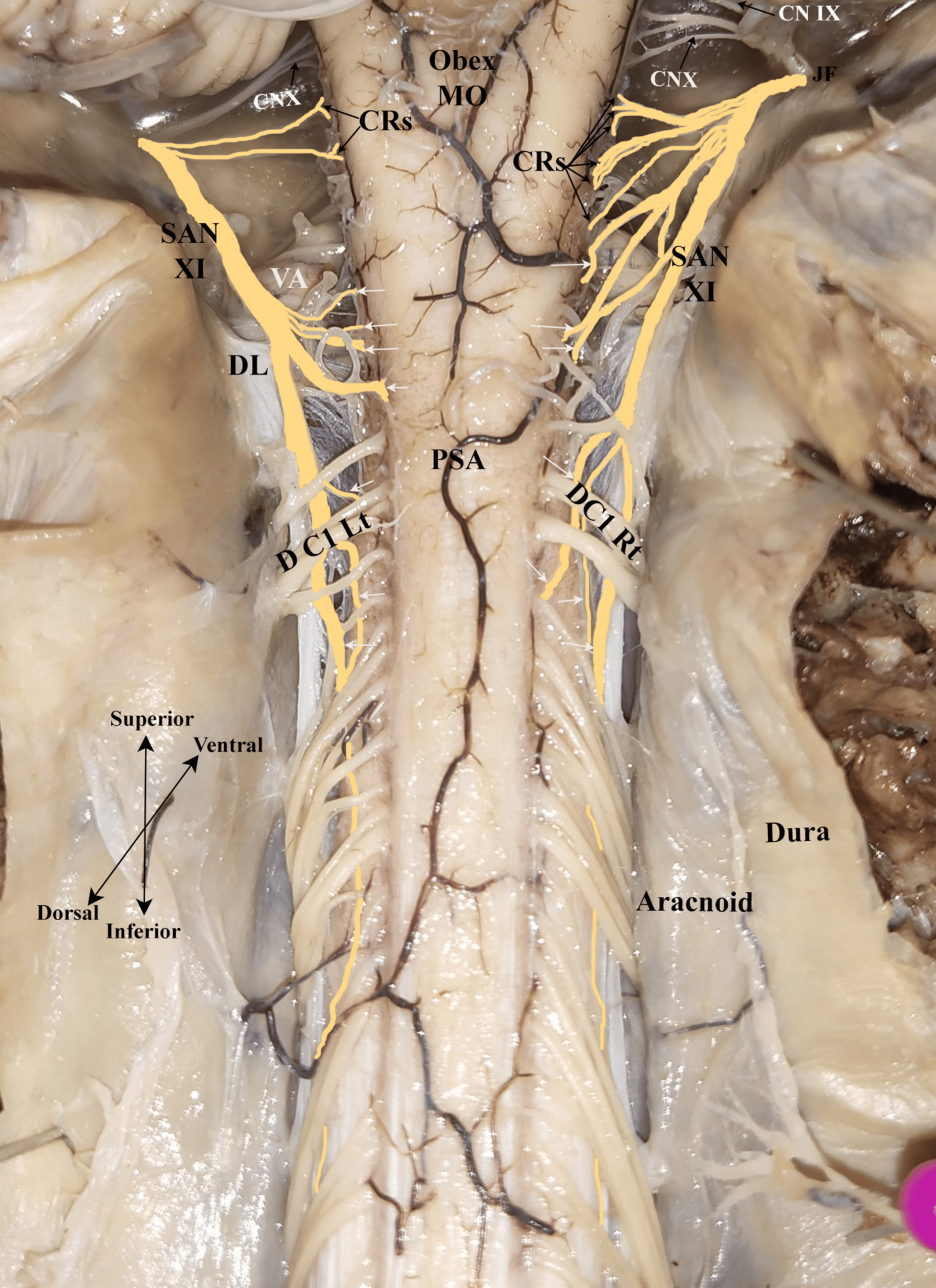
Spinal accessory nerve course from the cervical spinal segment to the jugular foramen (dorsal view). The black arrow indicates the cranial root, and the white arrow indicates the spinal root of the spinal accessory nerve. SAN XI, spinal accessory nerve; CRs, cranial roots; PSA, posterior spinal artery; D C1 Rt, dorsal first cervical spinal root on right side; D C1 Lt, dorsal first cervical spinal root on left side; MO, medulla oblongata; CN IX, glossopharyngeal nerve; CN X, vagus nerve; DL, denticulate ligament; JF, jugular foramen; VA, vertebral artery

A total of 30 cadavers (15 men and 15 women) were examined for bilateral SAN lengths. The SAN courses posterior to the vertebral artery and denticulate ligament (Figure 1). The length of the spinal root of the SAN was measured from the caudal origin of the cervical segment to the jugular foramen, where it connects with the cranial root to constitute a common trunk of the accessory nerve. The descriptive values for each side and sex are presented in Table 1 and Table 2.

**Table 1:** Spinal accessory nerve length on both sides. SD, standard deviation; CI, confidence interval

Parameter	Right spinal accessory nerve (n = 30)	Left spinal accessory nerve (n = 30)
Mean ± SD (mm)	56.8 ± 17.0	58.5 ± 13.1
Range (mm)	20-100	27-76
95% CI	50.4-63.2	53.7-63.4
Normality (Shapiro-Wilk)	p = 0.35	p = 0.41
Side comparison	t = 0.89; p = 0.38	

**Table 2:** Sex-wise comparison of spinal accessory nerve length. SD: standard deviation

Side	Sex	Mean ± SD (mm)	Range	P-value
Right	Male (n = 15)	63.3 ± 17.8	30-100	0.03
	Female (n = 15)	50.3 ± 13.8	20-69	
Left	Male (n = 15)	61.2 ± 12.7	31-75	0.27
	Female (n = 15)	55.9 ± 13.3	27-76	

The mean SAN length was 56.8 ± 17.0 mm on the right side and 58.5 ± 13.1 mm on the left side (n = 30 each). The SAN length ranges from 20 to 100 mm on the right side and from 27 to 76 mm on the left side. The side differences passed normality testing (Shapiro-Wilk, p = 0.35), and a paired t-test demonstrated no statistically significant side difference (t = 0.89; p = 0.38) (Table 1).

**Figure 2:**
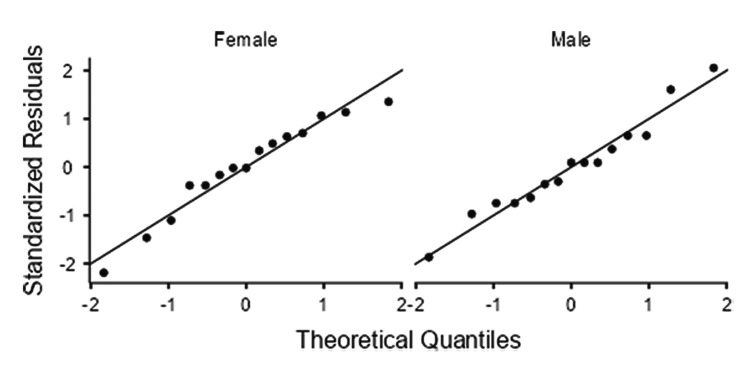
Right-side Q-Q plots of standardized residuals for SAN length in women (left) and men (right). SAN: spinal accessory nerve

**Figure 3:**
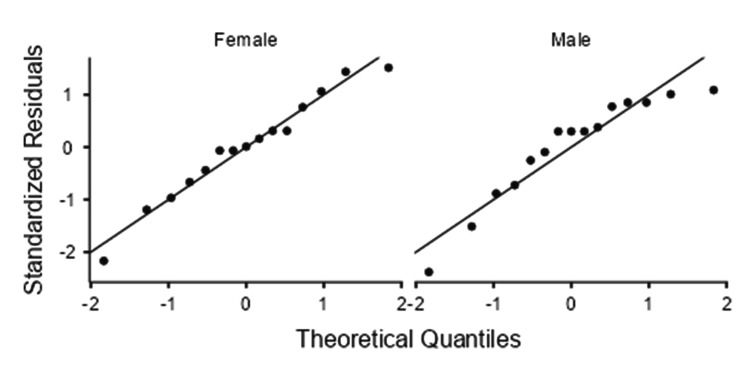
Left-side Q-Q plots of standardized residuals for SAN length in women (left) and men (right). SAN: spinal accessory nerve

Sex-wise analysis showed that the right SAN was significantly longer in men (63.3 ± 17.8 mm) than in women (50.3 ± 13.8 mm) (p = 0.034; Cohen’s d = 0.82), whereas the left side was 61.2 ± 12.7 mm in men and 55.9 ± 13.3 mm in women, with no significant sex difference (p = 0.27) (Table 2). In both men and women, the Q-Q plots of the standardized residuals demonstrated a close alignment of the data points along the theoretical reference line. No substantial curvature or tail deviation was observed, indicating that the SAN length values for both sex groups were approximately normally distributed (Figures 2, 3).

**Figure 4:**
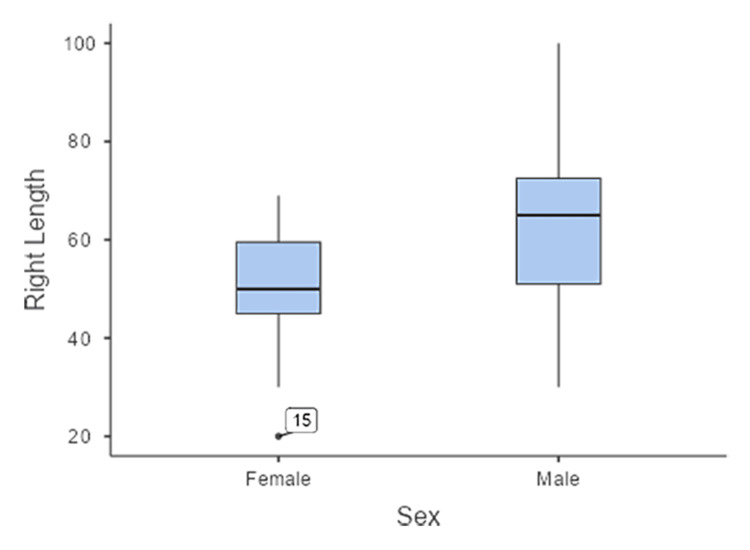
Boxplot showing the sex-wise distribution of the right SAN length. Men exhibited a higher median and greater variability than women, who exhibited a more compact distribution with one lower outlier. The point labelled “15” represents specimen number 15, which exhibited an unusually low SAN length (20 mm) on the right-side female and was identified as an outlier in the dataset. SAN: spinal accessory nerve

**Figure 5:**
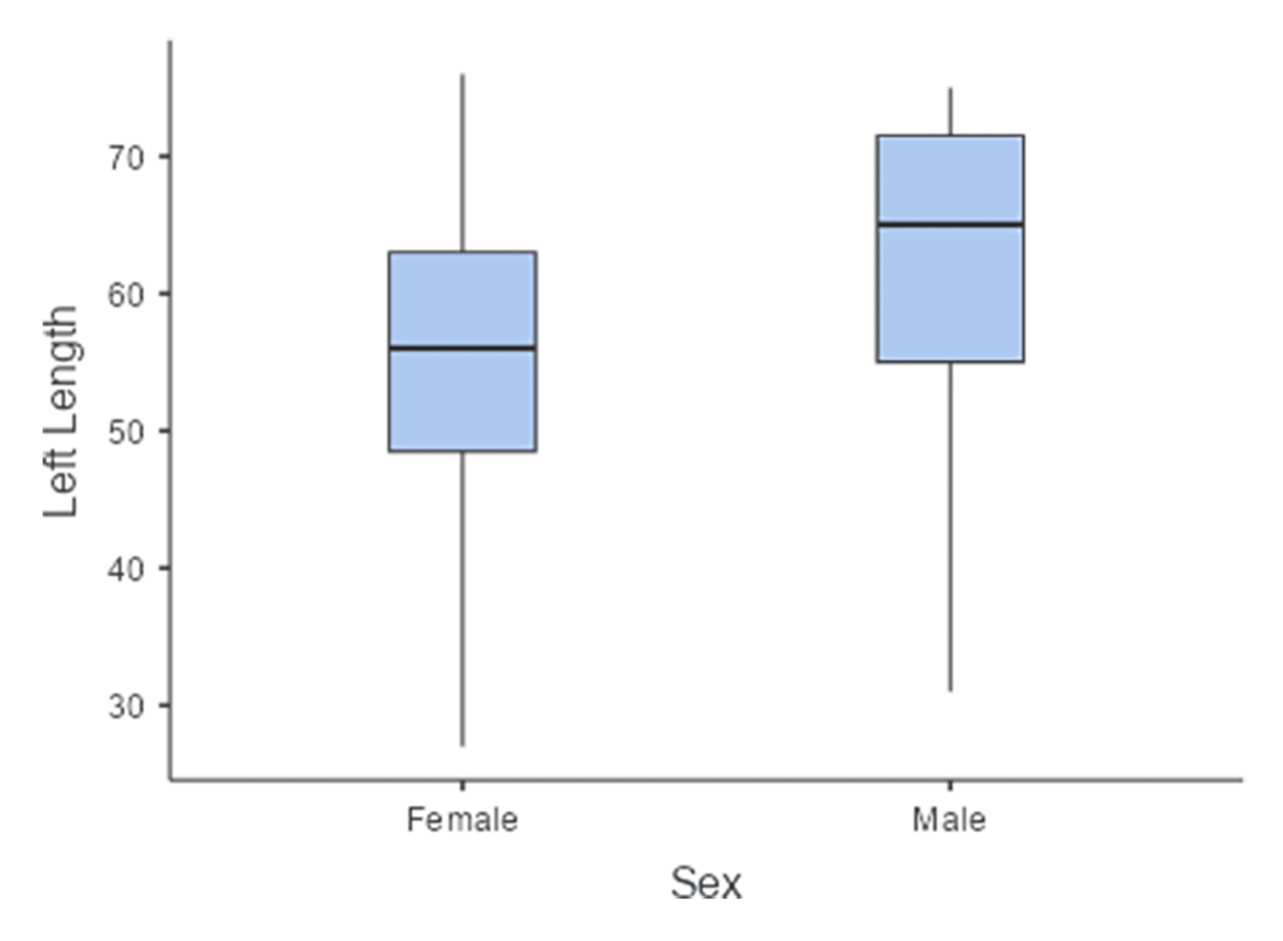
Boxplot showing the sex-wise distribution of the left SAN length. Men exhibited a higher median and greater variability than women, who exhibited a more compact distribution with one lower outlier. SAN: spinal accessory nerve

Sex significantly affected SAN asymmetry (right-left), with men showing greater asymmetry than women (p = 0.044; d = 0.77). The sex-wise boxplot revealed a clear difference in the distribution of the right SAN length. Female cadavers demonstrated a median value of approximately 50 mm, with an interquartile range (IQR) concentrated between approximately 47 and 58 mm, and one marked outlier at approximately 25 mm. Male cadavers showed a higher median of approximately 65 mm, with a broader IQR ranging from approximately 55 to 75 mm. The whiskers indicated a wider overall range in men, extending up to nearly 95 mm in length. These findings suggest that the right SAN tends to be longer in men than in women, with greater variability in the male group (Figure 4). The sex-wise distribution of the left SAN length showed notable differences. Female cadavers demonstrated a median left SAN length of approximately 56 mm, with an interquartile range (IQR) of approximately 50-62 mm. Male cadavers exhibited a higher median of approximately 65 mm and a wider IQR of approximately 55-72 mm. The whiskers extended more broadly in men than in women, indicating greater variability in SAN length in men. Overall, the left SAN was longer in men than in women (Figure 5).

The most caudal segment of the origin of the SAN was most frequently found at C5, observed in 33.3% of specimens, followed closely by C4 in 31.7%. Contributions from C3 were also frequent (21.7%). More caudal origins were less frequent, with C6 contributing to 6.6% and C7 in only 1.7% of cases. Very cranial caudal origins were rare, with C1 and C2 accounting for 1.7% and 3.3% of the cases, respectively.

**Figure 6:**
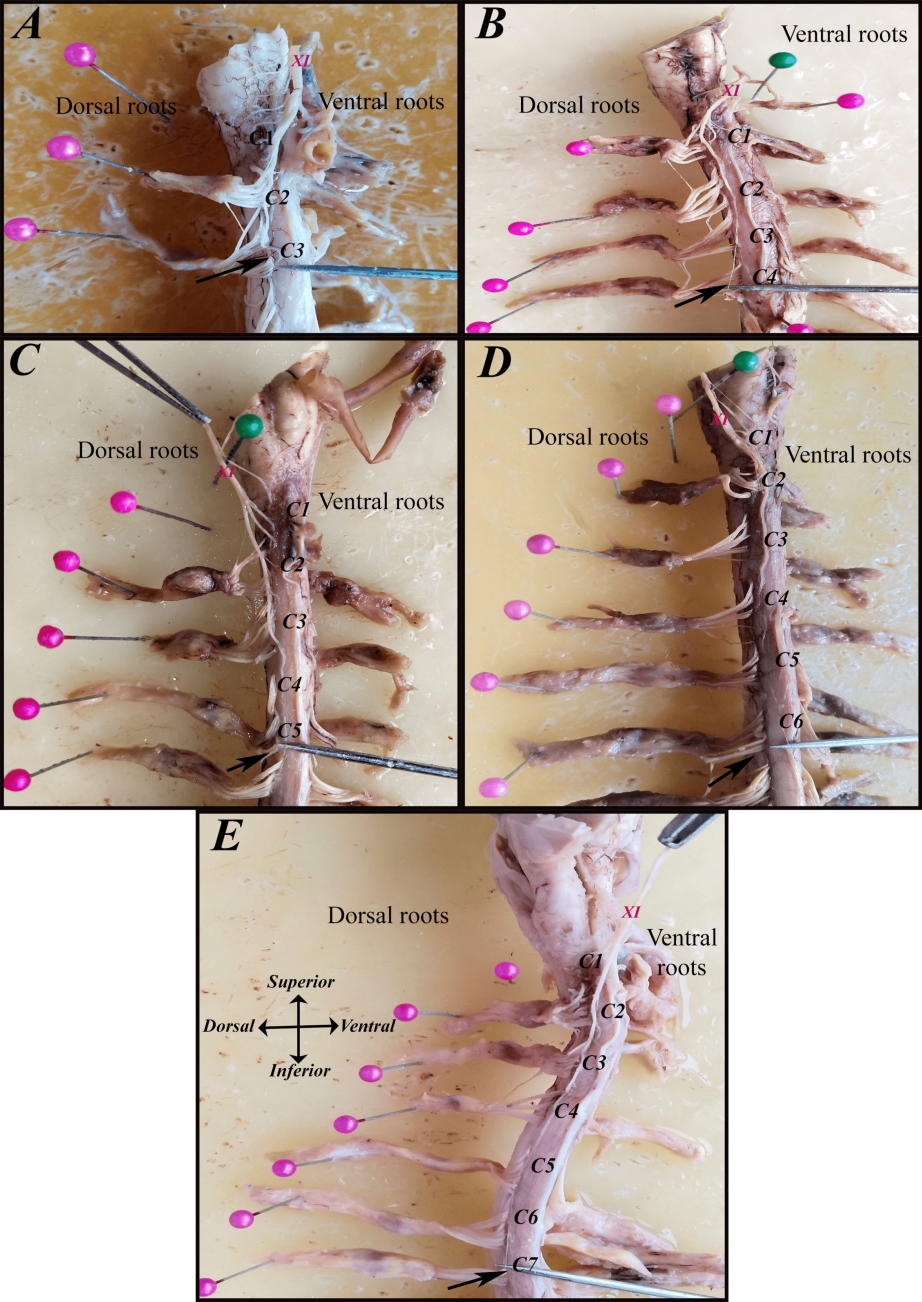
The most caudal segment from which the spinal accessory nerve (SAN {XI}) receives its rootlets (black arrows) on the right side in men: (A) C1-C3, (B) C1-C4, (C) C1-C5, (D) C1-C6, and (E) C1-C7.

**Figure 7:**
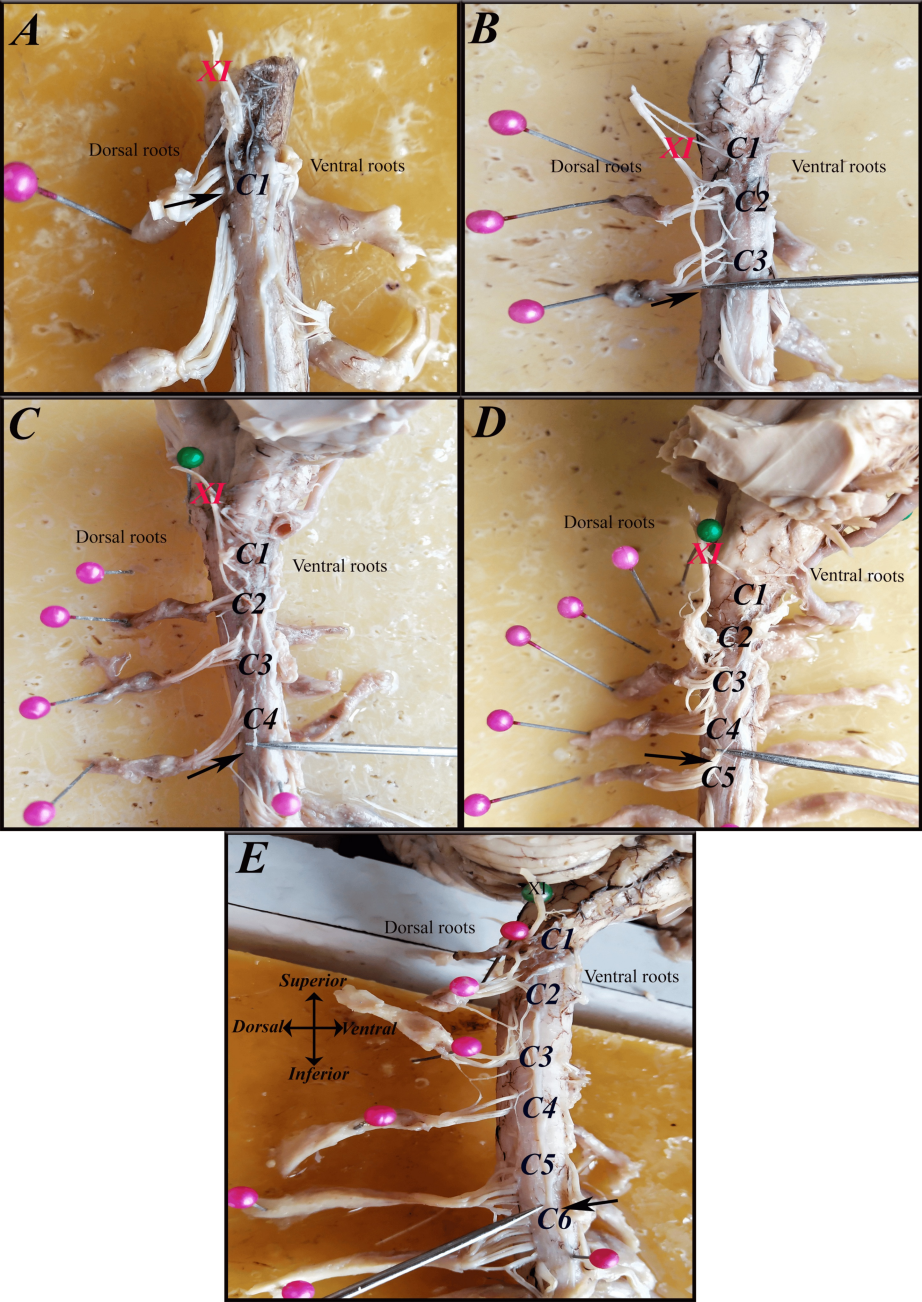
The most caudal segment from which the spinal accessory nerve (SAN {XI}) receives its rootlets (black arrows) on the right side in women: (A) C1, (B) C1-C3, (C) C1-C4, (D) C1-C5, and (E) C1-C6.

**Figure 8:**
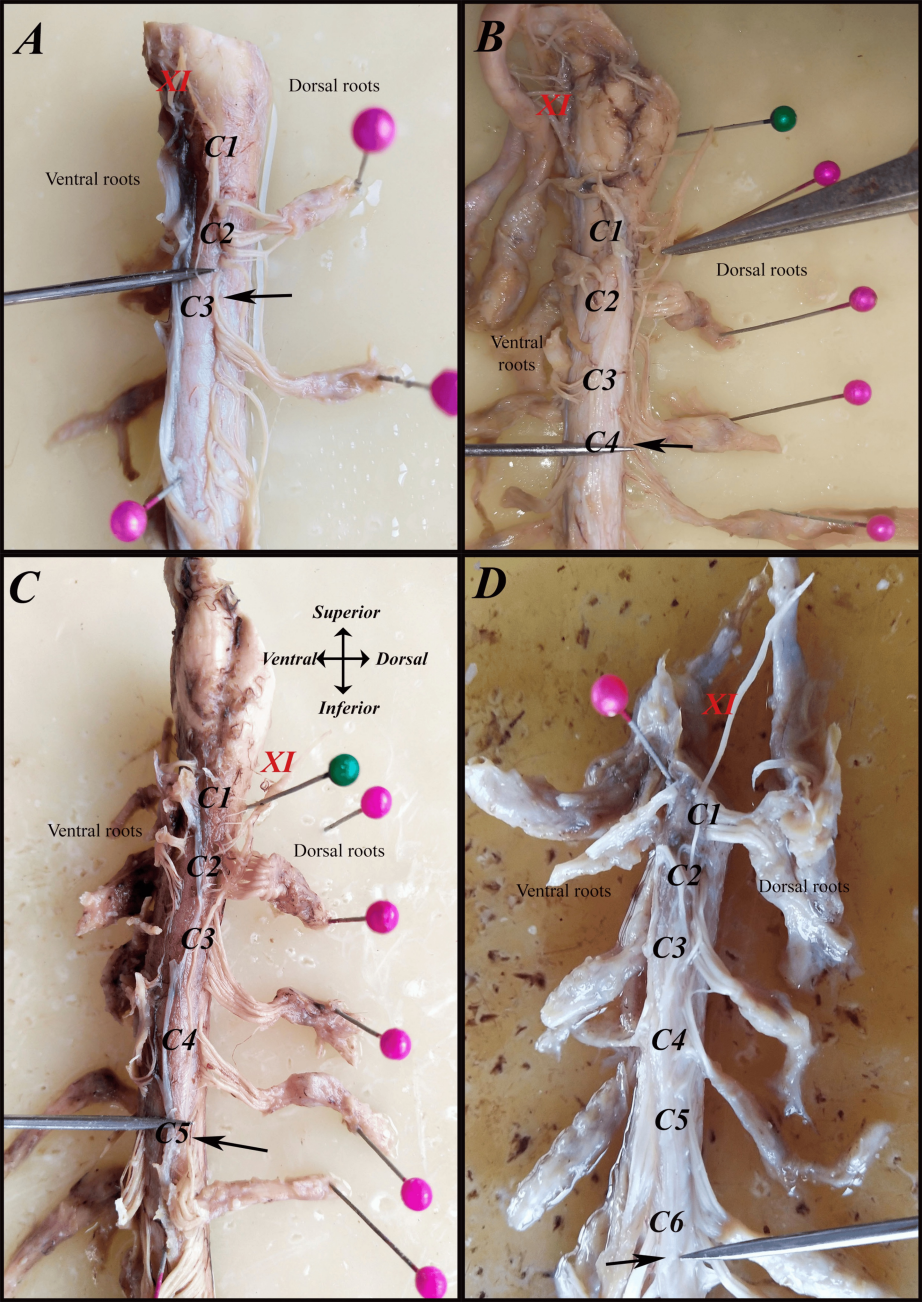
The most caudal segment from which the spinal accessory nerve (SAN {XI}) receives its rootlets (black arrows) on the left side in men: (A) C1-C3, (B) C1-C4, (C) C1-C5, and (D) C1-C6.

**Figure 9:**
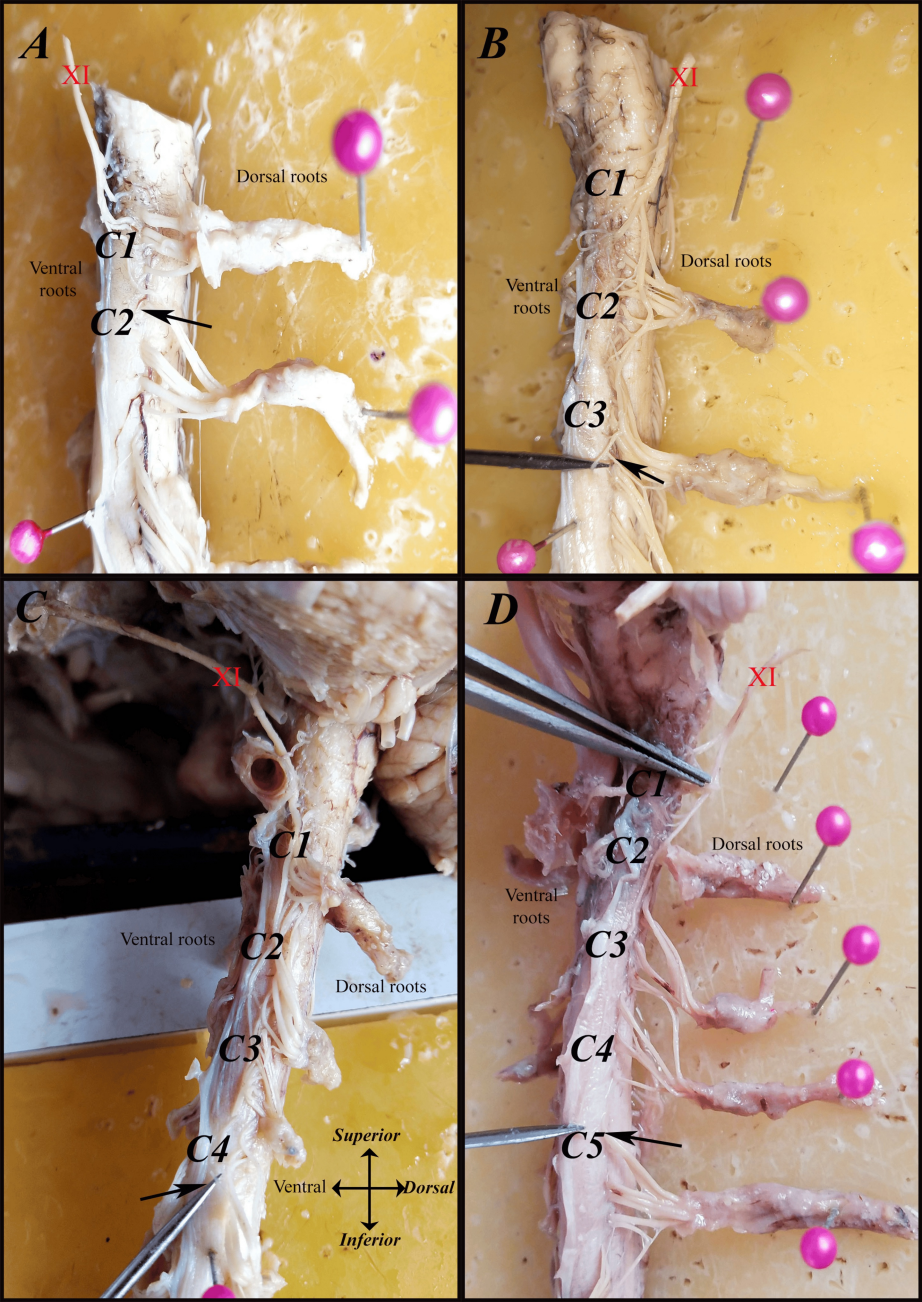
The most caudal segment from which the spinal accessory nerve (SAN {XI}) receives its rootlets (black arrows) on the left side in women: (A) C1-C2, (B) C1-C3, (C) C1-C4, and (D) C1-C5.

A sidewise comparison showed that the right side most frequently terminated at C4 (36.7%), followed by C3 (26.7%) and C5 (20%). On the left side, however, C5 was the predominant caudal contributor (46.7%), indicating a left-sided tendency for lower caudal extension. These findings highlight both interindividual and right-left variability in the caudal extent of the SAN rootlet origin (Table 3) (Figures 6-9).

**Table 3:** The most caudal segment that gives the rootlet in the formation of the spinal accessory nerve.

Most caudal segment of the origin of the spinal accessory nerve	Number of specimens on the right side (n = 30)	Number of specimens on the left side (n = 30)	Total number of specimens (n = 60)
C1	1 (3.3%)	0 (0%)	1 (1.7%)
C2	0 (0%)	2 (6.7%)	2 (3.3%)
C3	8 (26.7%)	5 (16.7%)	13 (21.7%)
C4	11 (36.7%)	8 (26.7%)	19 (31.7%)
C5	6 (20%)	14 (46.7%)	20 (33.3%)
C6	3 (10%)	1 (3.3%)	4 (6.6%)
C7	1 (3.3%)	0 (0%)	1 (1.7%)

The spinal segments that provide rootlets for the formation of the SAN were found in the C1-C7 segments. On the right, the most common cervical contribution pattern was C1-C4 (36.7%), followed by C1-C3 (26.7%) and C1-C5 (20.0%). On the left, C1-C5 predominated (46.7%), with C1-C4 (26.7%) and C1-C3 (16.7%). The chi-square test comparing the segmental pattern distribution between the sides was not statistically significant (χ² = 9.37; p = 0.15) (Table 4).

**Table 4:** The spinal segments from which the spinal accessory nerve received rootlets. Side comparison: χ² = 9.37, and p = 0.15.

Spinal segments that give rootlets in the formation of the spinal accessory nerve	Right side			Left side		
	Male (n = 15)	Female (n = 15)	Total (n = 30)	Male (n = 15)	Female (n = 15)	Total (n = 30)
C1	0 (0%)	1 (6.7%)	1 (3.3%)	0 (0%)	0 (0%)	0 (0%)
C1-C2	0 (0%)	0 (0%)	0 (0%)	0 (0%)	2 (13.3%)	2 (6.6%)
C1-C3	4 (26.7%)	4 (26.7%)	8 (26.7%)	3 (20%)	2 (13.3%)	5 (16.7%)
C1-C4	5 (33.3%)	6 (40%)	11 (36.7%)	5 (33.3%)	3 (20.1%)	9 (26.7%)
C1-C5	3 (20%)	3 (20%)	6 (20%)	6 (40%)	8 (53.3%)	14 (46.7%)
C1-C6	2 (13.3%)	1 (6.7%)	3 (10%)	1 (6.7%)	0 (0%)	1 (3.3%)
C1-C7	1 (6.7%)	0 (0%)	1 (3.3%)	0 (0%)	0 (0%)	0 (0%)

Dorsal rootlets contributing to SAN formation varied even at the same segmental level, depending on the specimens, with a maximum of six and a minimum of one on the right side and a maximum of five and a minimum of one on the left side at C2. Across all 60 nerves, the highest total (350) dorsal rootlet contribution arose from C2 (38.3%), followed by C3 (22.6%) and C1 (19.7%), respectively. The contributions from C5 to C7 were minimal (Table 5).

**Table 5:** Dorsal roots give rootlets in the formation of the spinal accessory nerve.

Dorsal roots	Rootlets (percent contribution) (out of 350 dorsal rootlets)
C1	69 (19.7%)
C2	134 (38%)
C3	79 (22.6%)
C4	41 (11.7%)
C5	21 (6%)
C6	5 (1.4%)
C7	1 (0.3%)

The mean total number of dorsal rootlets forming each SAN was 5.87 ± 2.89 on the right and 5.80 ± 2.61 on the left, with no significant difference between sides (Wilcoxon signed-rank test, p = 0.91) (Table 6).

**Table 6:** Dorsal rootlets per spinal accessory nerve on both sides. SD: standard deviation

Side	Mean ± SD	Range	P-value
Right (n = 30)	5.87 ± 2.89	1-15	0.91
Left (n = 30)	5.80 ± 2.61	2-13	

## Discussion

Understanding the length of the SAN is potentially beneficial for neurosurgeons because of its variability in the most caudal segment of origin of the spinal component of the SAN. The mean SAN length on the right side was 56.8 ± 17.0 mm, with men exhibiting a mean length of 63.3 ± 17.8 mm and women 50.3 ± 13.8 mm. On the left side, the mean length was 58.5 ± 13.1 mm, with men measuring 61.2 ± 12.7 mm and women measuring 55.9 ± 13.3 mm. The right side exhibited a range of 20-100 mm, whereas the left side ranged from 27 to 76 mm, highlighting the variability in SAN lengths, a phenomenon documented exclusively in our study. Our SAN length measurements could influence treatment strategies for patients experiencing postoperative pain, potentially leading to more precise management, which warrants further investigation. Previous research has established that the accessory nerve originates solely from the upper cervical spinal cord segments, with no part arising from the medulla. These results challenge traditional teachings but align with the early descriptions of the accessory nerve [4,22].

Nevertheless, cranial roots were observed in all specimens. Our findings support the classical view that the SAN is mainly composed of spinal rootlets from the upper cervical spinal cord. The spinal root of the accessory nerve emerges from a motor nucleus extending from the spinal cord-medulla junction to the sixth cervical segment. Textbooks indicate that this motor nucleus spans the C1-C6 segments [8]. In our specimens, the accessory nerve received rootlets from the C1-C7 segments in various distributions, with no rootlets found below the C7 segment. The most common segment was C5, followed by C4, C3, C6, C2, C1, and C7, consistent with Oliveira et al.’s findings [15]. In both men and women, the right side involved C1-C4, while the left side involved C1-C5. These results emphasize the variability in the formation of the accessory nerve, differing from previous reports, which noted the most caudal spinal rootlets ranging from C1-C6 [10] to C2-C5 [12], to C3-C7 [14], with C3 [12] and C4 [10,14] being the most frequent levels (Table 7). No rootlets were detected in the T1 segment, and the rootlet distribution varied across the specimens [16]. The greater involvement of C1-C4 and C1-C5 in SAN formation reflects the patterns described in modern microsurgical studies by Tubbs et al., who emphasized the complexity of cervical root contributions and their surgical significance [6].

**Table 7:** Comparison to other studies of the most caudal segment of the origin of the spinal accessory nerve. SAN: spinal accessory nerve

Authors	Sample size	The most caudal segment of the origin of SAN						
		C1	C2	C3	C4	C5	C6	C7
Hagenah et al. (1983) [14]	100	-	-	23%	52%	23%	1%	1%
Oliveira et al. (1985) [15]	50	4%	10%	14%	22%	26%	20%	4%
Oh and Chung (2008) [10]	50	2%	5%	32%	30%	22%	9%	-
Saylam et al. (2009) [12]	49	-	8.1%	42.9%	32.7%	16.3%	-	-
Present study	60	1.7%	3.3%	21.7%	31.7%	33.3%	6.66%	1.7%

This study presented the average number of the dorsal rootlets of the accessory nerve at each cervical segment and demonstrated a higher number of the rootlets at the more cranial C2 (2.3), followed by C3 (1.3), C1 (1.2), C4 (0.6), C5 (0.3), C6 (0.2), and C7 (0.03). These findings differ somewhat from those of Oh and Chung [10] and Kim et al. [21], who reported 4.4 and 1.6 at C1, 3.1 and 2.3 at C2, 2.4 and 2.2 at C3, 0.8 and 0.9 at C4, and 0.4 and 0.3 at C5, respectively. The predominant contribution of C2 observed in this study aligns with the embryological descriptions by Pearson, who documented early neural crest development influencing the formation of the SAN [13]. Additionally, our results correlate with those of Hagenah et al., who reported variability in the location and density of accessory nerve motoneurons [14]. Furthermore, C2 has consistently been recognized as the dominant segment and dorsal root contributing to SAN formation, likely due to its robust motor neuron pool. The clinical implications of SAN variability are significant, as iatrogenic SAN injury remains one of the most frequent complications of neck dissection. Modern studies by Restrepo et al. [20], Kim et al. [21], and Tubbs et al. [23] have emphasized the consequences of SAN injury on trapezius function, shoulder elevation, and scapular stabilization. Acquaintance with the SAN length, segmental contribution, and rootlet origin is essential for minimizing morbidity during posterior cervical triangle dissections, selective neurotomy, and skull base tumor resections. Compared to previous studies, this investigation provides population-specific data for Indian cadavers, addressing the gap in regional anatomical literature. The moderate variability observed in this study is consistent with global findings, suggesting that SAN morphology is evolutionarily conserved but exhibits individual fluctuations attributable to developmental factors.

Limitations

This study was limited by the use of formalin-fixed cadaveric specimens, which may not fully replicate the in vivo neural dimensions or elasticity. The cross-sectional design of this study did not permit the evaluation of developmental or age-related changes in SAN morphology.

## Conclusions

Previous literature has not reported a detailed measurement of the accessory nerve length. In this study, the SAN was measured as 56.8 ± 17.0 mm on the right side and 58.5 ± 13.1 mm on the left side, with no significant difference observed between the two sides. The SAN receives rootlets from cervical segments C1-C7. We believe that our findings, which exhibit some differences from those of previous studies regarding accessory nerve formation, underscore the importance of dorsal rootlet contributions. This information is crucial for surgical planning and nerve repair procedures in the neck region, as it aids in minimizing iatrogenic injury to the SAN and optimizing functional preservation during procedures such as neck dissection and reconstructive surgery.
